# Supplementary figures and images for: Is metformin use associated with low mortality in patients with type 2 diabetes mellitus hospitalized for COVID-19? a multivariable and propensity score-adjusted meta-analysis
Source: PLoS One. 2023 Feb 23;18(2):e0282210. doi: 10.1371/journal.pone.0282210 (PMC9949644; doi:10.1371/journal.pone.0282210)

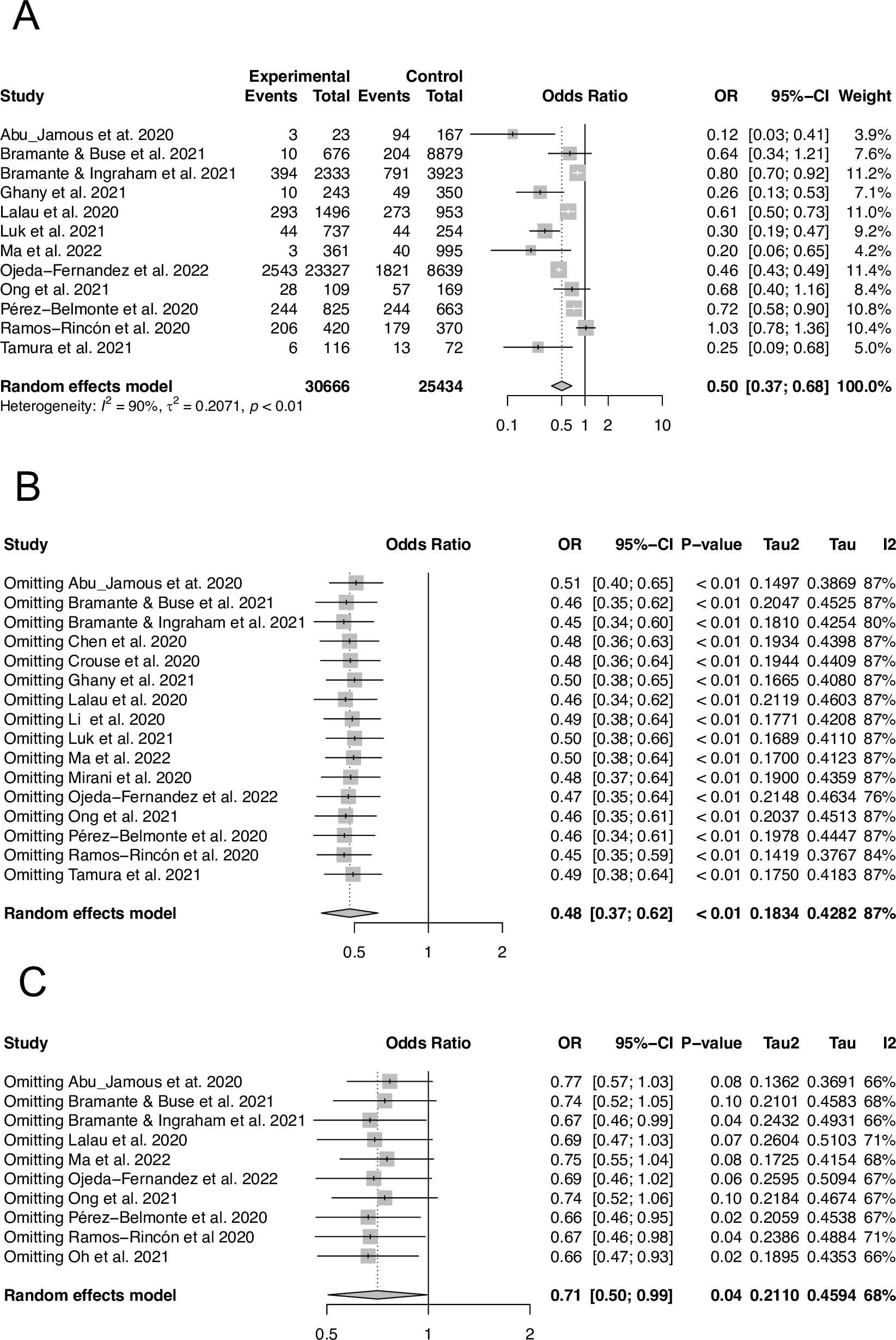

Supplement: S1 Fig — (TIF) [file pone.0282210.s002.tif]
