# Supplementary material for: Is metformin use associated with low mortality in patients with type 2 diabetes mellitus hospitalized for COVID-19? a multivariable and propensity score-adjusted meta-analysis
Source: PLoS One. 2023 Feb 23;18(2):e0282210. doi: 10.1371/journal.pone.0282210 (PMC9949644; doi:10.1371/journal.pone.0282210)
Supplement: Attachment — Submitted filename: Response to reviewers.docx [file pone.0282210.s003.docx]

<https://mynet.slhn.org/~/media/Images/MyNet/Logos/Easton/Easton-logo-blue.ashx?la=en>

250 S. 21^st^ Street

Easton, PA 18042

Nov. 27th, 2022

Dear Dr. Hirst,

We appreciate the interest in our manuscript. We would like to thank the reviewers for their comments. We have revised our manuscript (PONE-D-21-37827) according to reviewer’s comments. Please find a point-by-point response to reviewers’ concerns below. The manuscript has been carefully edited.

1. “Please ensure that your manuscript meets PLOS ONE's style requirements, including those for file naming.” “please add line numbers to help reviewers”

Response: We have revised our manuscript according to PLOS ONE’s style requirements and added the line numbers.

2. “The main finding of this paper is that although unadjusted analysis suggests a significant benefit of metformin, there is no effect of metformin on mortality after adjustment for confounders. This should be highlighted in the discussion, rather than the significant finding of the unadjusted analysis. Amend this in the abstract and main body of paper.”

Response: We updated search and have performed new analysis. The results have been included in the section of Results and discussion.

3. “The Prospero page needs updating as it indicates that the authors are still at the search stage.”

Response: We have updated the status in the PROSPERO.

4. “Searches: These only identified a very small number of studies. Was a medical librarian consulted to assist with searches? Please provide full details of the search strategy, as the described searches would have missed articles. Consider including terms such as covid*, death, biguanide*, and metformin brand names.

Were terms for diabetes included in the search?”

Response: We did not consult librarian for assisting with the search. We did not include the keyword diabetes in the search. We manually screened diabetic patients in the studies after identification. Since our search was more than 1 year ago, we performed updated search using the expression “((SARS-CoV-2) OR (Covid-19)) AND ((Metformin) OR (Biguanides) OR (Fortamet) OR (Glumetza) OR (Glucophage) OR (Biomet)) AND ((Mortality) OR (Death))” as suggested. Three new studies have been included for analyses.

5. “In the meta-analysis of adjusted data, please give details of methods used to allow odds ratio and hazard ratios to be combined. Were they assumed to be the same? If this is the case, then I would expect sensitivity analyses to be carried out to assess the impact of these approximations.”

Response: Raw event data extracted from individual study were pooled using the Mantel-Haenszel approach and effect size (OR, HR) was pooled using the inverse-variance method. This has been described in the methods. We also included sensitivity analyses in Figure 2 and 3.

6. “Outpatient metformin use and in-hospital mortality Page 6 – the following sentence needs some clarification: “There were 15 studies with or without attempt to adjust for confounding examining the association between outpatient metformin use and in-hospital mortality”

What is meant by with or without adjusting? Is this a repeat of the numbers in the previous paragraph?”

Response: We have revised the text.

7. “The results section contains methods and interpretation – please ensure that methods are in the methods section and interpretation is moved to the discussion leaving results only to be reported in the results”

Response: We have revised the results.

8. “Include details of quality assessment scores for each of the included studies and risk of bias.”

Response: the Newcastle-Ottawa Scale scores have been included in Table 1.

9. “This needs to be structured, starting with the key findings.

Discuss risk of bias.”

Response: We have revised the section of discussion and discussed more on bias.

Reviewer #1:

1. “What would be of particular interest is to be the first to draw some lessons from rather robust studies. This consists in fact of – to my knowledge – seventh meta-analysis.”

Response: Thank you for your comments. Of note, we performed this study and submitted it more than a year ago. In this study, we aimed to address whether outpatient (prior to admission) or inpatient metformin (during hospitalization) therapy offers low in-hospital mortality in patients with type 2 diabetes mellitus hospitalized for COVID-19 especially by multivariable and propensity score-adjusted effect sizes (OR and HR). We discussed the issues in the previously published meta-analysis in the second paragraph of the section of the discussion. We think our study is interesting.

2. “We are speaking about observational studies, not about interventional studies. Therefore the words “decrease” (mortality), as early as in the title, and “reduction”, appearing numerous times in the text, are not appropriate.”

Response: Thank you for your comments. We agree that we did a meta-analysis of observational studies. We have revised our title. But we believe metformin therapy is interventional and treatment, so “decrease” and “reduction is related to metformin therapy not to study types.

3. “The terms “outpatients” and “inpatients” are also inappropriate. The question should rather be phrased as whether or not the putative beneficial association between metformin treatment and COVID outcomes is due to metformin treatment prior to hospital admission, its continuation during the hospital stay, or both. Therefore the wording would be: “metformin discontinued”, “metformin continued”, “maintenance of metformin”, etc. (during a hospital stay).”

Response: Thank you for your suggestion. We have specifically clarified “outpatient” and “inpatient” metformin use in the introduction.

4. “what means “A Multivariable and Propensity Score-adjusted Meta-analysis?””

Response: Meta-analysis of studies with adjusted effect size derived from multivariable regression or propensity score matching.

5. “The wording is often weak (one example, in the abstract: “Random effect models were applied for meta-analysis due to variation among studies”. In fact, the issue is not that of “variations” (between studies). Rather it is as following: the inclusion of a large number of covariates does not compensate for a lack of key variables (such as BMI, the estimated glomerular filtration rate, duration of diabetes, etc.). Given that, the studies with a low sample size, with a low number of covariates, and with a lack of the major covariates should not even be considered. It is already impossible to compare studies with either 172 or more than 2 million people with diabetes!”

Response: Thank you for your comments. We have revised the text. The random-effects model is conventionally used to address between-study heterogeneity. We did unadjusted and adjusted analyses to address other bias. we excluded studies with the number of subjects less than 50.

6. “There are just a few words about the putative protective effects for metformin; this is clearly not enough (what about effects on microvascular blood flow and pericytes, microvascular permeability, hemostasis, glycocalyx, hydrogen sulfide, ER stress, etc.?).”

Response: We have revised the text and added the protected effects on microvessels.

7. “The first lines of the Discussion do not deal with the main results.”

Response: We have revised the section of discussion.

8. “To put it simply, the compilation of short and/or weak studies cannot lead to a firm conclusion. And, as it happens, the by far largest study (more than 2 million people: Ojeda-Fernandez, Diabetes Obesity Metab 2022) is lacking.”

Response: We completed our search before Oct 1^st^, 2021 and submitted our paper on Nov. 29^th^, 2021. Now We completed new search and have included the above study along with 2 more studies.

9. “Given all these difficulties, the limitations subsection is far too weak.”

Response: We have revised the limitation subsection.

Reviewer #2:

1. “You state that you assessed bias and refer to internal to analysis confounding issues as well as publication bias, but you do not characterize the latter. What were your systematic bias assessment results across the studies?”

Response: We have performed the analyses of publication bias using funnel plots and Egger’s tests. We also included the Newcastle-Ottawa Scale scores in Table 1.

2. “Heterogeneity is quite high across pooled analyses. This should be discussed. For instance, (1) how was A1C and complicated diabetes accounted across studies; and, (2) in which studies was metformin already part of the patients' regimen versus added while in house or once diagnosis with SARS-CoV-2 was accomplished.”

Response: We have revised the limitation subsection.

3. “The discussion and conclusion language should acknowledge more directly (depending upon your response to the previous questions) that metformin may indicate lower risk because being managed for diabetes... currently, your presented data and analyses does not support a direct effect and yet you close the article expressing only a caveat of pooled analyses statistical significance.”

Response: We have performed new analyses with more studies. We have revised the results and conclusions.

Sincerely,

Mahesh Krishnamurthy, MD

Department of Internal Medicine,

St Luke’s University Health Network-Easton Campus

Phone: 484-658-0692

Email: Mahesh.Krishnamurthy@sluhn.org
