# Supplementary material for: Is metformin use associated with low mortality in patients with type 2 diabetes mellitus hospitalized for COVID-19? a multivariable and propensity score-adjusted meta-analysis
Source: PLoS One. 2023 Feb 23;18(2):e0282210. doi: 10.1371/journal.pone.0282210 (PMC9949644; doi:10.1371/journal.pone.0282210)
Supplement: Attachment — Submitted filename: Response to reviewers.docx [file pone.0282210.s004.docx]

<https://mynet.slhn.org/~/media/Images/MyNet/Logos/Easton/Easton-logo-blue.ashx?la=en>

250 S. 21^st^ Street

Easton, PA 18042

Feb. 4th, 2023

Dear reviewers,

We would like to thank you for your comments. We have revised our manuscript (PONE-D-21-37827R1) according to your comments. Please find a point-by-point response to your concerns below. The manuscript has been carefully edited.

Reviewer #1:

1. “I must admit that I am not very interested in meta-analyses but I would also highlight the great work done by the authors for improving their manuscript.

I agree with all modifications but one: as long as studies are observational, it is never possible to speak about “reduction” (of mortality – because of metformin) (lines 21, 158, 211, 266, 269…), and even less about a “trend toward a reduction” (L. 158: non-scientific)”

Response: Thank you for your comment. As you pointed out that this is a meta-analysis of observational studies, we have revised the text and made it clear throughout the study that there is an association between metformin use and hospital mortality, but not direct causation. The word “reduction” is used as a noun or adjective not as a verb which may imply direct causation.

Reviewer #2:

1. “Consider doing a quick sensitivity analysis where you remove all studies that have a 6 or lower Newcastle-Ottowa score (indicating high risk). Those studies are substantially smaller than the studies that you scored more highly and so a single study removal method for sensitivity analysis would likely be insufficient to discern the effect by class”

Response: We have added additional analysis regarding this (Supp fig. 1A).

2. “The bigger issue for me is that the manuscript is written in a way that suggests that metformin was applied for the purpose of COVID-19 therapy. That seems unlikely, particularly in the one category where you observed a multi-variate statistically significant effect. This matters in how readers (even technically grounded ways) understand the role of metformin here. You also seem to avoid talking about DM management in your background as a reason for exploring the effect. This needs to be addressed through-out. The Limitations section additions relevant to my earlier comments are appropriate. What I am talking about now is how the question is framed and the discussion and conclusions are proffered. I suspect that you simply have taken for granted that readers will understand this difference, but you do not state it, and your language suggests direct therapeutic application for the purpose of COVID-19 in a milieu where it has been among several problematic narratives avoiding more rounded approaches to patient management. This can be stated succinctly in the intro, discussion, and conclusion sections and easily corrected.”

Response: Thank you for your comment. We have revised the text.

Sincerely,

Mahesh Krishnamurthy, MD

Department of Internal Medicine,

St Luke’s University Health Network-Easton Campus

Phone: 484-658-0692

Email: Mahesh.Krishnamurthy@sluhn.org
